# Development of an Integrated Virtual Reality System with Wearable Sensors for Ergonomic Evaluation of Human–Robot Cooperative Workplaces

**DOI:** 10.3390/s22062413

**Published:** 2022-03-21

**Authors:** Teodorico Caporaso, Stanislao Grazioso, Giuseppe Di Gironimo

**Affiliations:** Fraunhofer Joint Lab IDEAS, Department of Industrial Engineering, University of Naples Federico II, 80125 Naples, Italy; stanislao.grazioso@unina.it (S.G.); giuseppe.digironimo@unina.it (G.D.G.)

**Keywords:** virtual reality, wearable sensors, ergonomic analysis, cooperative workplace, human–robot physical interaction

## Abstract

This work proposes a novel virtual reality system which makes use of wearable sensors for testing and validation of cooperative workplaces from the ergonomic point of view. The main objective is to show, in real time, the ergonomic evaluation based on a muscular activity analysis within the immersive virtual environment. The system comprises the following key elements: a robotic simulator for modeling the robot and the working environment; virtual reality devices for human immersion and interaction within the simulated environment; five surface electromyographic sensors; and one uniaxial accelerometer for measuring the human ergonomic status. The methodology comprises the following steps: firstly, the virtual environment is constructed with an associated immersive tutorial for the worker; secondly, an ergonomic toolbox is developed for muscular analysis. This analysis involves multiple ergonomic outputs: root mean square for each muscle, a global electromyographic score, and a synthetic index. They are all visualized in the immersive environment during the execution of the task. To test this methodology, experimental trials are conducted on a real use case in a human–robot cooperative workplace typical of the automotive industry. The results showed that the methodology can effectively be applied in the analysis of human–robot interaction, to endow the workers with self–awareness with respect to their physical conditions.

## 1. Introduction

In human–robot cooperation scenarios, humans and robots perform different tasks that should be fulfilled simultaneously in terms of time and space [1]. Regarding the workers’ safety, many efforts have been applied in order to identify safety areas for the worker to avoid possible collisions [2,3]. In addition, several works also pay attention to on the optimization of the task allocation in order to reduce the execution costs required to the worker [4]. However, in this scenario workers may also have to deal with repetitive tasks, which can become an issue for their health [5]. To quantify the human risk factors, a lot of methods and tools have been developed for ergonomics assessment. They are usually divided into four categories [6]: self–report, observational tools, virtual simulations (where digital human models are constructed and their activities are simulated [7]), and direct measurements (where real data are collected using motion capture system and/or wearable sensors [8]). In the last category, direct measurements using wearable sensors can be effectively used in real workplaces. The use of inertial measurement units (IMU) allows the assessment of typical ergonomic indices, such as rapid upper limb assessment (RULA), rapid enter body assessment (REBA), posture evaluation index (PEI), workcell evaluation index (WEI), Ovako working posture analyzing system (OWAS), and European Assembly Work-Sheet (EAWS), as presented in different works [7,9,10]. In addition, it has been demonstrated that significant biomechanical and physiological changes in fatigued muscles can be assessed using surface electromyography (sEMG) signals [11]. In this context, a wearable sensors system composed of IMU and sEMG sensors has also been used in real time to monitor workers by measuring muscular efforts and postures for the evaluation in parallel with typical ergonomic indices such as RULA [12].

Although this ergonomic analysis can also be carried out in real time, the possibility to have a continuous interaction with the industrial environment is very poor. In this context, a possible strategy is to use human–in–the–loop virtual reality (VR) technologies. Indeed, these technologies guarantee the accuracy of ergonomic analysis by incorporating human–in–the–loop in the simulation of a realistic environment. For this aim, consumer VR devices such as VR headsets (e.g., HTC Vive, Oculus Rift, and Samsung Gear VR) provide the users with an immersive experience [13]. The usability of these technologies can be evaluated using different subjective multidimensional measures to assess the workload, such as NASA Task Load Index (NASA–TLI) or System Usability Scale (SUS) [14]. These interactive technologies can be used to try different workplace configurations and, at the same time, to give the worker real–time feedback about his ergonomic status during the execution of the task. In this field, recently, the authors of [15] developed a VR platform on Unity 3D, an open-source software with models of collaborative robot (i.e., cobot), enabling kinematic modeling and control of robots using tracked controllers. Another possible open–source software useful for robotic simulation in VR is CoppeliaSim. Indeed, the CoppeliaSim VR Toolbox provides a set of tools to experience robot simulation in VR and to return user interactions [16].

The literature presents several VR systems with the integration of the ergonomic evaluation. For example, in [17], the authors presented a VR system for ergonomic analysis in aircraft assembly using as VR platform Unity 3D with Oculus sensor as VR device. The ergonomic analysis was performed using MS Kinect data and the ergonomic evaluation was based on RULA and REBA scores. However, the system did not offer the possibility of a real–time evaluation. Another interesting example is the VR–Ergo log system [18], an immersive VR system (developed using HTC Vive) combined with an IMU system (integrated with heart rate monitoring) that allows to obtain an ergonomic assessment. The data collected with the IMU system are used to move a digital human model (Jack from Siemens) and are processed in real time with the assessment of RULA, OWAS, OCRA, and lifting index. However, the system did not allow the acquisition of EMG data. This latter aspect has been underlined by the authors in [18] as future work worth investigating.

In summary, although it is well established within the ergonomics discipline that muscular effort can lead to discomfort and injuries [19], none of the presented VR systems show a real–time ergonomic assessment based on muscular activity evaluation. In this context, the main contribution of our VR system is to show, in real time, the EMG–based ergonomic evaluation within the immersive virtual environment. For this aim, we propose a VR system that combines the use of wearable sensors for a real–time assessment of cooperative workplaces from the ergonomic point of view. To this end, we used the robotic simulator CoppeliaSim in combination with sEMG sensors and an accelerometer. The proposed approach has been tested in a real use case of a cooperative workplace, which consists of a pick–and–place task in the automotive industry.

## 2. System Architecture

**Figure 1:**
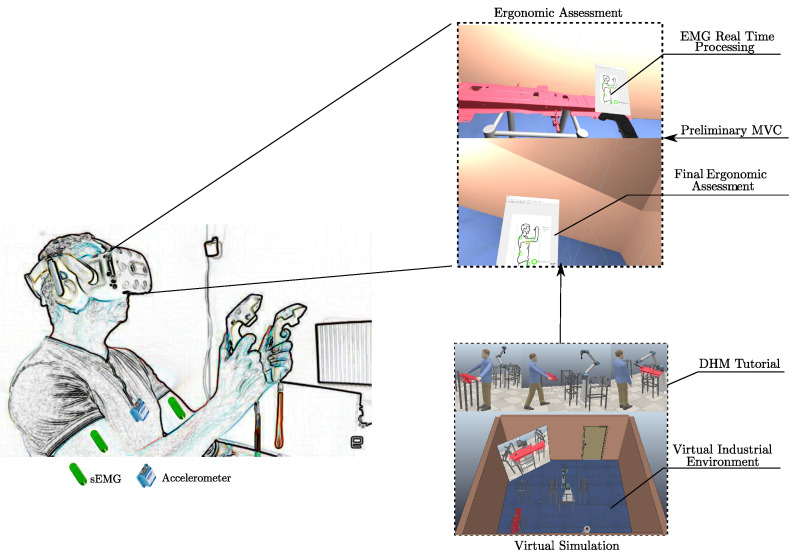
A schematic representation of the system architecture. On the left, an image of a real scenario: the user wears the wearable sensors (i.e., sEMG and accelerometer) and VR devices (i.e., headset and controllers). On the bottom–right, the block of “Virtual Simulation”, composed of a photosequence of the DHM tutorial (**top**) and an image of the virtual environment (**bottom**). On the top-right, the block of the “Ergonomic Assessment” with the input data: the virtual simulation and the “Preliminary MVC”. In the block on the top, there is an image in virtual simulation with the “EMG Real Time Processing” and on the bottom is an image of the “Final Ergonomic Assessment”.

Figure 1 shows the architecture used in this work. In our system, we use a mixed–prototyping strategy involving VR environment, computer–aided design (CAD) objects, and human subject [20,21]. The real industrial environment is reconstructed in the robotic simulator CoppeliaSim. In particular, the objects of the virtual environment are created using a standard CAD software (i.e., CATIA) while the cobot is directly found in the CoppeliaSim model library. In the simulation, the user’s interaction with the environment is guaranteed by consumer virtual reality devices (i.e., headset, tracking system, and controllers). In addition, an associated tutorial through a digital human model (DHM) is implemented (first sequence of images in the block “virtual simulation” in Figure 1). For the ergonomic assessment, a user is asked to perform all the tasks required by industrial use case. A tool for the real–time acquisition and processing of biosignals through wearable sensors is developed in Matlab environment (block “Ergonomic Assessment” in Figure 1). In detail, the ergonomic assessment is performed using multiple sEMG sensors placed on a specific user’s muscle and one accelerometer located at the end of the vertebral column. Preliminary data related to maximum voluntary contraction (MVC) of the worker are required for a correct evaluation. The output of the ergonomic analysis, i.e., the “EMG Real Time Processing” and the “Final Ergonomic Assessment” (at the end of the task), are then shown within the immersive environment in real time. In the next two sections, the methodology used to realize the virtual reality simulations and ergonomic assessment are presented.

## 3. Virtual Reality Simulations

In this section, the steps implemented for the construction of the virtual environment of the simulation and the related DHM tutorial are presented.

### 3.1. Virtual Environment

The virtual simulations are built using the robotic simulator CoppeliaSim combined with SteamVR. The HTC–Vive system is employed to allow the human immersion and interactions with the simulated environment. The Steam VR is used to visualize the simulation in VR and to track the state of each component of the HTC–Vive. The environment is developed with positioning the objects in the same way as the real work environment. During the virtual simulation, the feedback from the ergonomic analysis (printed according to the processing described in the next section) is shown inside the VR simulation, through a specific functionality of SteamVR. More specifically, a window is attached to one of the controllers and can be seen by the user while performing other operations (as shown in Figure 1). Finally, the “Final Ergonomic Assessment” is shown when the worker completes the task.

### 3.2. DHM Tutorial

In order to allow the user to better understand task steps, an immersive tutorial based on the use of DHM was developed. In this way, the workers can familiarize themselves with the task for a better execution in first–person. In this way, it is possible to collect realistic ergonomic data by wearable sensors. The setup of the scene is the same developed for the virtual simulation. This simulation is carried out using a DHM in which the movements of the human and the cobot are programmed according to the requests of the industrial task (see the block in Figure 1).

## 4. Ergonomic Assessment

**Figure 2:**
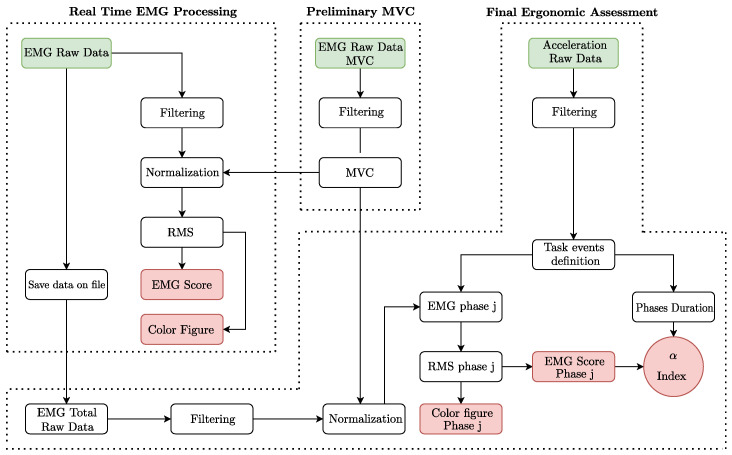
A flowchart showing all the steps implemented in the ergonomic analysis.

In this section, the operations defined for the ergonomic assessment in real–time and in post–processing are explained in detail. The sEMG sensors and an accelerometer are employed to acquire, respectively, muscle activation and kinematic data for biomechanical events assessment during the virtual reality simulation. The data processing, implemented in Matlab environment starting from the data collected with wearable sensors, is schematized in Figure 2. It is divided into three main sections (see dashed lines in Figure 2): preliminary MVC, real–time EMG processing, and final ergonomic assessment. The most important outputs of each section are contained in red blocks, as can be seen in Figure 2.

### 4.1. Preliminary MVC

This is a preliminary section which takes place before the real–time acquisition. In this phase, the user’s MVCs are acquired and filtered with a moving average filter and a Butterworth low–pass filter. These specific parameters are defined according to the characteristics of the industrial task. The final output is to obtain the MVC for each muscle under investigation, which will be used during the real-time processing and for the final ergonomic assessment in order to normalize the sEMG signals.

### 4.2. Real Time EMG Processing

**Figure 3:**
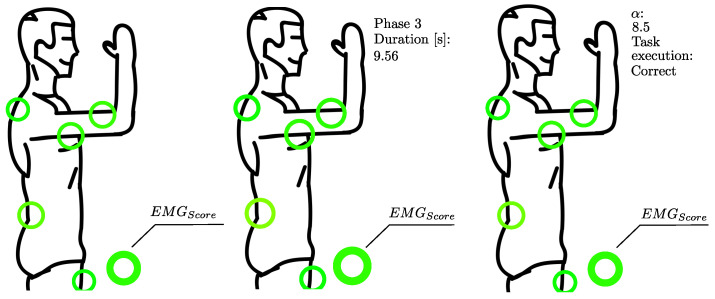
(**Left**) Example of image shown during the real-time acquisition; (**Center**) example of image shown during the final ergonomic assessment related to a generic phase; (**Right**) example of image related to the assessment of the whole task.

The purpose of this section is to collect and to process, in real time, the data from the sEMG sensors. This data processing is applied each second during the execution of the task. The processing starts with the same filtering operation described in the previous subsection. Then, using the MVC values previously obtained, the signals are normalized. Starting from the normalized sEMG values, the following operations are carried out: (i) calculation of RMS values; (ii) definition of a color based on RMS values; (iii) printing a specific image to show the level of muscle activation on the body (see Figure 3); (iv) definition of the $EMG_{\mathrm{Score}}$, an average of the RMS values; and (v) saving data on a .txt (which will be the input of the final ergonomic assessment). For the point (ii), in order to obtain a specific color for each RMS value, we consider the RGB color space, in which all the colors are obtained from the combination of the red (R), green (G), and blue (B) colors. Thus, the definition of the color related to each index is carried out according to the following system of equations:

(1) $$\begin{array}{c} \left\{\begin{array}{c} R=5.1\cdot \mathrm{RMS}\\ G=255\;\mathrm{RMS}\leq 50\\ B=0 \end{array}\right. \end{array}$$

or:

(2) $$\begin{array}{c} \left\{\begin{array}{c} R=255\\ G=255-5.1\cdot (\mathrm{RMS}-50)\;\mathrm{RMS}>50\\ B=0 \end{array}\right. \end{array}$$

The points (iii) and (iv) are the main outputs of this section. Indeed, once the colors are determined they are plotted on an image (showing a human body shape, see Figure 3) through a circle for each muscle and a circle for the $EMG_{\mathrm{Score}}$. The $EMG_{\mathrm{Score}}$ assessment is obtained according the following equation:

(3) $$EMG_{\mathrm{Score}}=\frac{1}{n}\sum_{i=1}^{n}{\mathrm{RMS}}_{i}$$

where *i* is the generic index that represents the single muscle and *n* is the total number of muscles involved in the ergonomic evaluation.

### 4.3. Final Ergonomic Assessment

This section starts as soon as the time specified for the real-time acquisition ends. Its main purpose is evaluating a synthetic ergonomic index for the specific phase of the task. For this aim, a set of phases are defined from the the identification of specific events on the center of mass (CoM) vertical acceleration graph of the whole task. In addition, in order to delete the phase shift, signals are filtered two times (in both the directions). Thus, also for this section, the sEMG signals are filtered and normalized, as descried in the previous subsection. Then, the RMS value and the $EMG_{\mathrm{Score}}$ (with the related color) are assessed and, finally, a specific image is printed for each phase of the task. The last output of this section is a synthetic index, called α, that is a weighted average of the $EMG_{\mathrm{Score}}$ evaluated for the single phases. It is defined according the following equation:

(4) $$\alpha =\sum_{i=1}^{m}EMG_{{\mathrm{Score}}_{i}}\cdot \left(\frac{t_{i}}{T_{tot}}\right)$$

where *i* is the generic index that represents a single phase of the task, $t_{i}$ is the time duration of the generic phase *i*, *m* is the number of the phases composing the task, and $T_{tot}$ is the total time of the whole task.

## 5. Experiments

In this section, the proposed methodology exposed is applied in a real industrial use case. This use case is part of the Integrated and COllaborative Systems for the smArt Factory (ICOSAF) project, which aims to develop and integrate technologies and solutions for a collaborative factory with a growing integration of the operator with collaborative automation systems.

### 5.1. Use Case Description and Implementation

The use case of this work involves a human–robot workplace where the cobot is represented by a UR10 robot. The device automatically checks the quality of welds, through big data elaborations by a computer, and gives to the worker information about the quality of the process and about the maintenance status. The cobot checks the quality of the welds through an ultrasound tool placed as the end–effector. The work cycle hypothesized for the use case is divided into the following steps:

- Pickup of a metal component from the load stand. The component is an assembly of metal parts that arrives to the control spot already assembled. It weighs 3.4 kg.
- Manual transport of the component to the robot stand.
- Wait until the robot analyzes each one of the 50 welding points on the component.

According to the overall work cycle description, the human task can be divided into the following phases: (i) bend and reach plus grasp (BRG): the phase in which the worker leans to pick up the component; (ii) arise from bend, get (ABG): the phase in which the worker stands up while holding the component; (iii) turn body and walk (TBW): the phase in which the worker turns and walk to the examination stand while holding the component; (iv) bend and reach (BR): it is another leaning phase in which the user leans to place the component on the robot stand; (v) positioning (*P*): the user takes some time to place the component while he is leaning; (vi) arise from bend, put (ABP): the phase in which the worker stands up not holding the component.

In order to recreate the virtual environment according to the system architecture exposed in Section 2 in CoppeliaSim, the industrial use case was built with all objects required. In addition, the robot with an appropriate end–effector was able to move on the welds control points. A DHM included in the CoppeliaSim library is programmed to simulate the task for the development of the virtual tutorial. In addition, to make the training more effective, some panels with extra instructions about the task were created and programmed to appear during specific moments of the work cycle simulated by the DHM (e.g., when the DHM picks up the component or when he presses the button). The same scenario, without the DHM, was used for VR simulations where there is the possibility to interact with the objects in the scene using HTC–Vive controllers. In particular, for the user’s interaction, different functions were programmed: (i) to pick the component; (ii) to start robot examination; (iii) to help the user to put the component in the correct position. Finally, an additional feature was implemented to help the user in the positioning of the component on the examination stand based on the collision detection mechanism of CoppeliaSim.

### 5.2. Experimental Setup

The experiments were conducted in the MARTE laboratory of the University of Naples Federico II (Fraunhofer Joint Lab IDEAS—Cesma). The laboratory is equipped with an HTC-Vive system composed of a headset, two base stations, and two controllers. The base stations were mounted on two tripods with an inclination of 45${}^{\circ }$ downward. They were positioned in the opposite corners of a square defining a tracking area of 25 m^2^. The experiments involved a male volunteer, 166 cm tall, with body mass of 62 kg, who had to replicate the task described in Section 5.1 while wearing wearable sensors.

According to the goal exposed in Section 4, the wearable sensors chosen were part of the Bitalino Revolution Board Kit. It is a biosignals acquisition wearable board equipped with Bluetooth, with the possibility to connect at the same time up to six sensors. For the proposed methodology, five sEMG sensors (dynamic range ±1.64 mV; sample frequency: 1000 Hz) placed on specific muscles and one uniaxial accelerometer (dynamic range: ±3 g; sample frequency: 1000 Hz) placed at the bottom of the vertebral column at L5 level (approximately the height of CoM) were used.

The muscles to be monitored were chosen after carrying out a comparison with similar tasks, according to literature [22] and after a research on muscles activation in response to specific movements, such as as the SENIAM project [23]. The muscles considered were the following: biceps brachii (BB), long head of the triceps brachii (TB), anterior deltoid (AD), erector spinae at L3 level (ES), and rectus femoris (RF). To place the electrodes on the selected muscles in the correct positions, the indications from the SENIAM project were followed [23]. Before moving on with the real-time acquisition, the MVC for each muscle had to be collected to perform normalization on the EMG signals. For this aim, specific exercises suggested by SENIAM for each muscle under investigation were carried out. According to the characteristics of the industrial task, the following filter parameters were applied: moving average filter with a time window of 150 ms and a Butterworth low-pass filter fourth-order with a cut-off frequency of 2 Hz [22]. For the acceleration, a fourth-order Butterworth low–pass filter was applied with a cut-off frequency of 2 Hz (six times the frequency of stride, according to [24]). In order to make the Bitalino Revolution Board wearable, a 3D case specifically made for the board was 3D printed. It was attached to the user’s belt, so that he could easily drag it around during the acquisition. In addition, in order to give to the user the weight feedback of the handled metal component, he was equipped with two weights fixed on his wrists. In this way, actual responses from the sEMG sensors were obtained. The weights used are 2 kg each, which approximate the weight of the real metal component.

### 5.3. Experimental Results

**Figure 4:**
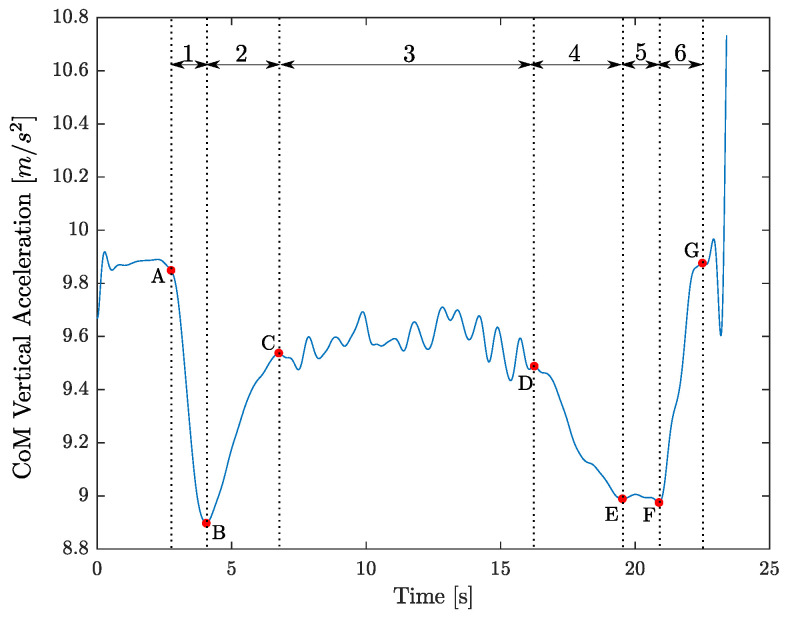
Vertical acceleration of the CoM as function of the time. In red circles, the notable points correspond to the different events. Notice that, different to definitions in the section, we have used the following nomenclature: 1 = BRG; 2 = ABG; 3 = TBW; 4 = BR; 5 = *P*; 6 = ABP.

The industrial task was replicated by the voluntary. The phases of the whole task, individuated in Section 5.1, were defined and described in relation to the vertical CoM acceleration graph through the identification of notable points related to the specific events (Figure 4):

- **BRG**: The BRG phase is defined as the time that elapses between the first instant of the worker bending in front of the load stand, called event A, and the instant in which the worker grasps the metal component (called event B). The BRG timing is so expressed equal to
  (5) $$BRG=t_{B}-t_{A}$$
  where $t_{B}$ is the first temporal instant of the minimum of the vertical CoM acceleration under a threshold (fixed equal to ϵ = 0.9 g); $t_{A}$ is assessed starting from the point B, coming back as the previous first temporal instant of the maximum.
- **ABG**: The ABG phase is defined as the time that elapses between the event B and the instant in which the worker returns to standing up straight (called event C). The ABG timing is so expressed equal to
  (6) $$ABG=t_{C}-t_{B}$$
  where $t_{C}$ is assessed starting from the point B as the following first temporal instant of the maximum.
- **TBW**: The TBW phase is defined as the time that elapses between the event C and the first instant of the worker bending in front of the robot (called event D). The TBW timing is so expressed equal to
  (7) $$TBW=t_{D}-t_{C}$$
  where $t_{D}$ is assessed starting coming back from the point E (related to $t_{E}$, that is the first temporal instant of the minimum of the vertical CoM acceleration under the threshold ϵ after the point C) as the previous first temporal instant of the maximum.
- **BR**: The BR phase is defined as the time that elapses between the event D and the event E (the instant in which the worker starts the positioning of the metal component on the robot stand). The BR timing is so expressed equal to
  (8) $$BR=t_{E}-t_{D}$$
- **P**: The *P* phase is defined as the time that elapses between the event E and the instant in which the worker ends the positioning of the metal component on the robot stand (called event F). The *P* timing is so expressed equal to
  (9) $$P=t_{F}-t_{E}$$
  where $t_{F}$ is assessed starting from the point E as the last temporal instant of the minimum under the threshold ϵ.
- **ABP**: The ABP phase is defined as the time that elapses between the event F and the instant in which the worker returns to standing up straight (called event G). The ABP timing is so expressed equal to
  (10) $$ABP=t_{G}-t_{F}$$
  where $t_{G}$ is assessed starting from the point F as the following first temporal instant of the maximum.

**Figure 5:**
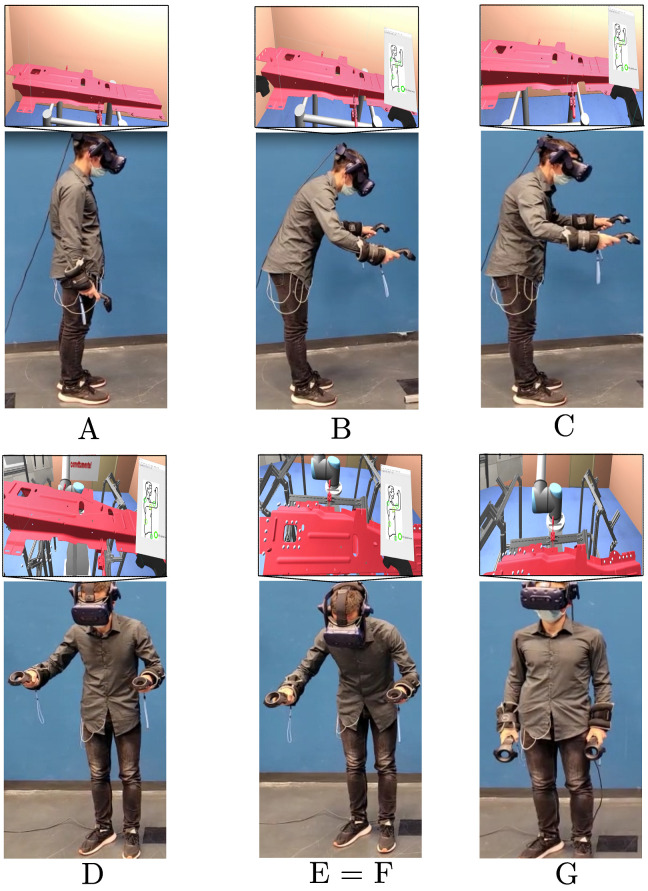
The events of the real task, and the associated view in the virtual environment (placed above in the block over the related real figure), corresponding to (**A**) beginning of BRG phase, (**B**) end of BRG phase: grasping event, (**C**) end event of the ABG, (**D**) beginning event of BR phase, (**E**=**F**) events during the *P*, (**G**) end event of the ABP phase. Notice that events E and F are both represented in the same picture, because the subject is in the same position.

According to events defined on the vertical CoM acceleration graph (see Figure 4), the related highlights from the real task and from the virtual task are shown in Figure 5. A summary of the results collected for each phase is reported in Table 1. According to these values, the obtained α index was equal to 8.5.

## 6. Discussion

The final ergonomic assessment obtained with the proposed system (shown in Table 2) underlines how the task under investigation is not very demanding for the user. These results are in accordance with a typical ergonomic evaluation carried out by virtual simulation using a digital human model (Jack, Siemens) that defined the constraints according to the use case characteristics (described in Section 5.1). Indeed, the ergonomic indices obtained for the fifth male percentile (which represents the volunteer’s characteristics) show values of no risk (i.e., RULA < 3 and REBA < 2) or low risk (i.e., RULA < 5 and REBA < 4). In addition, the PEI values are included in a non-critical range. Indeed, they are between 0.66 and 1.31 with none of the three terms assuming values above 1.

In addition, we also tested the usability of the proposed system. The evaluation of the task obtained using the NASA–TLI questionnaires, according to the data collected by the volunteer, is reported in Table 3. The “Weights” underline how “Mental Demand” and “performance” are considered as the most important variables, while “temporal demand” is the least important one. For the “Rating” assessment, “mental demand”, “physical demand”, and “effort” had the highest loading (i.e., between 25 and 30) that, in accordance with the literature, can be interpreted as a medium workload [25]. Finally, starting from the collected data, the global score of the NASA-TLI is equal to 24 (which can also be considered as a medium workload [25]).

### Limitations

Firstly, the sample size was very limited; indeed, the VR system was tested in only one scenario and with only one unskilled volunteer. Future works will be focused on testing the proposed system architecture with a statistically significant sample of industrial workers with different genders that cover a wide range of stature percentiles of the selected population. Despite this limitation, the main goal of this work, consisting of presenting a new sensor-based framework for VR, was achieved. Secondly, regarding the ergonomic evaluation, the main limitation is that our VR system does not consider classical ergonomic indices (such as RULA, REBA, and OWAS). Although for these indices a comparative evaluation in virtual simulation was carried out through Jack software, future development will consider the integration of an IMU sensor system to integrate the actual ergonomic analysis with RULA and REBA scores in the real-time ergonomic assessment. Despite this limitation, this work uses an established approach for ergonomic analysis through the use of sEMG sensors. Thirdly, regarding usability, an actual limitation of the proposed system is the users’ difficulties in wearing the sEMG sensors. This issue, also common for other sensors used in VR systems [26], can be overcome with the use of wearable clothes with textile electrodes [27].

## 7. Conclusions

In this work, we presented the definition of a virtual reality system composed of a robotic simulator (with headset and controllers) combined with the use of surface electromyography sensors and accelerometer for real-time testing and validation of cooperative workplaces. Then, we applied the system to a real industrial use case related to a human–robot task in the automotive industry. The results showed that a worker is able to understand and perceive his ergonomic status and safety conditions while he is directly performing the task in the immersive virtual environments. We report two evaluations: (1) quantitative evaluation, with respect to empirical methods for ergonomic assessment; (2) qualitative evaluation, in terms of usability of the proposed EMG-based VR system. With respect to the first evaluation, we can consider the EMG-based ergonomic indices valid. With respect to the second evaluation, we can consider that the global workload on the operator can be considered as medium. Future works can be focused on testing the proposed system with a statistically significant sample of industrial workers with different anthropometric characteristics, including multiple IMU to obtain more kinematic data, and using textile electrodes for sEMG sensors to improve the wearability of the system.

## Figures and Tables

**Table 1:** Root mean square of the normalized muscle activation values for the five muscles (BB: biceps brachii, TB: long head of the triceps brachii; AD: anterior deltoid, ES: erector spinae at L3 level, RF: rectus femoris) under investigation for each phase (BRG: bend and reach plus grasp; ABG: arise from bend, get; TBW: turn body and walk; BR: bend and reach; *P*: positioning; ABP: arise from bend, put) of the task with relative time duration (Time) and $EMG_{\mathrm{Score}}$.

Phase	Time [s]	BB [%]	TB [%]	AD [%]	ES [%]	RF [%]	*EMG*_Score_
*BRG*	1.31	10.9	9.1	3.5	18.4	1.9	8.8
*ABG*	2.69	7.5	9.1	3.5	21.6	1.9	8.7
*TBW*	9.56	8.3	8.9	2.9	25.9	2.9	9.6
*BR*	3.28	8.0	9.0	3.3	21.3	3.3	9.0
*P*	1.36	6.5	9.6	3.8	19.0	2.2	8.2
*ABP*	1.61	5.2	9.7	3.2	12.4	2.7	6.7
Overall	19.81	7.5	9.0	3.1	21.5	2.3	8.7

**Table 2:** RULA, REBA, and PEI scores for each phase of the task. Notice that in this assessment the phases BR and *P* are merged.

Phase	RULA [-]	REBA [-]	PEI [-]
BRG	3	2	1.03
ABG	2	1	0.80
TBW	2	2	1.12
BR+*P*	3	3	1.31
ABP	2	1	0.66

**Table 3:** NASA–TLI score.

Demand	Rating	Weight
Mental demand	30	4
Physical demand	25	2
Effort	25	2
Performance	6	4
Temporal demand	10	1
Frustration	15	2

## Data Availability

The data presented in this study are available on request from the corresponding author. The data are not publicly available due to privacy policy.

## Abbreviations

The following abbreviations are used in this manuscript:

VR	Virtual Reality
DHM	Digital Human Model
MVC	Maximum Voluntary Contraction
IMU	Inertial Measurement Units
RMS	Root Mean Square
RULA	Rapid Upper Limb Assessment
REBA	Rapid Entire Body Assessment
PEI	Posture Evaluation Index
WEI	Workcell Evaluation Index
OWAS	Ovako Working posture Analysing System
EWAS	European Assembly Work-Sheet
$t_{i}$	Time duration of the generic phase
$T_{tot}$	Total time of the whole task
sEMG	Surface electromyography
ICOSAF	Integrated and COllaborative Systems for the smArt Factory
BRG	Bend and Reach plus Grasp
ABG	Arise from Bend, Get
TBW	Turn Body and Walk
BR	Bend and Reach
*P*	Positioning
ABP	Arise from Bend, Put
BB	Biceps Brachii
TB	long head of the Triceps Brachii
AD	Anterior Deltoid
ES	Erector Spinae at L3 level
RF	Rectus Femoris
SUS	System Usability Scale
NASA TLI	NASA Task Load Index

## References

[1] Bonci A., Cen Cheng P.D., Indri M., Nabissi G., Sibona F. (2021). Human–robot perception in industrial environments: A survey. Sensors.

[2] Salmi T., Marstio I., Malm T., Montonen J. Advanced safety solutions for human–robot-cooperation. Proceedings of the ISR 2016: 47st International Symposium on Robotics.

[3] Pedrocchi N., Vicentini F., Matteo M., Tosatti L.M. (2013). Safe human–robot cooperation in an industrial environment. Int. J. Adv. Robot. Syst..

[4] Fusaro F., Lamon E., De Momi E., Ajoudani A. A human-aware method to plan complex cooperative and autonomous tasks using behavior trees. Proceedings of the 2020 IEEE-RAS 20th International Conference on Humanoid Robots (Humanoids).

[5] Shin H.J., Kim J.Y. (2007). Measurement of trunk muscle fatigue during dynamic lifting and lowering as recovery time changes. Int. J. Ind. Ergon..

[6] Battini D., Persona A., Sgarbossa F. (2014). Innovative real-time system to integrate ergonomic evaluations into warehouse design and management. Comput. Ind. Eng..

[7] Tarallo A., Di Gironimo G., Gerbino S., Vanacore A., Lanzotti A. (2019). Robust interactive design for ergonomics and safety: R-IDEaS procedure and applications. Int. J. Interact. Des. Manuf. (IJIDeM).

[8] Panariello D., Grazioso S., Caporaso T., Palomba A., Di Gironimo G., Lanzotti A. (2022). Biomechanical analysis of the upper body during overhead industrial tasks using electromyography and motion capture integrated with digital human models. Int. J. Interact. Des. Manuf. (IJIDeM).

[9] Caputo F., Greco A., D’Amato E., Notaro I., Spada S. (2018). Imu-based motion capture wearable system for ergonomic assessment in industrial environment. Proceedings of the International Conference on Applied Human Factors and Ergonomics, Orlando, FL, USA, 21–25 July 2018.

[10] Akhmad S., Arendra A., Findiastuti W., Lumintu I., Pramudita Y.D. Wearable IMU Wireless Sensors Network for Smart Instrument of Ergonomic Risk Assessment. Proceedings of the 2020 6th Information Technology International Seminar (ITIS).

[11] Lorenzini M., Kim W., De Momi E., Ajoudani A. A new overloading fatigue model for ergonomic risk assessment with application to human–robot collaboration. Proceedings of the 2019 International Conference on Robotics and Automation (ICRA).

[12] Peppoloni L., Filippeschi A., Ruffaldi E., Avizzano C. (2016). A novel wearable system for the online assessment of risk for biomechanical load in repetitive efforts. Int. J. Ind. Ergon..

[13] Hasan M., Perez D., Shen Y., Yang H. (2021). Distributed Microscopic Traffic Simulation with Human-in-the-Loop Enabled by Virtual Reality Technologies. Adv. Eng. Softw..

[14] Feick M., Kleer N., Tang A., Krüger A. The Virtual Reality Questionnaire Toolkit. Proceedings of the Adjunct Publication of the 33rd Annual ACM Symposium on User Interface Software and Technology.

[15] Hurtado C.V., Flores A.R., Elizondo V., Palacios P., Zamora G. Work-in-Progress: Virtual Reality System for training on the operation and programing of a Collaborative Robot. Proceedings of the 2021 IEEE Global Engineering Education Conference (EDUCON).

[16] Bogaerts B., Sels S., Vanlanduit S., Penne R. (2020). Connecting the CoppeliaSim robotics simulator to virtual reality. SoftwareX.

[17] Vosniakos G.C., Deville J., Matsas E. (2017). On immersive virtual environments for assessing human-driven assembly of large mechanical parts. Procedia Manuf..

[18] Daria B., Martina C., Alessandro P., Fabio S., Valentina V., Zennaro I. (2018). Integrating mocap system and immersive reality for efficient human-centred workstation design. IFAC-PapersOnLine.

[19] Haslegrave C., Corlett E., Wilson J.R., Corlett N. (2005). Work Condition and the Risk of Injuries.

[20] Ahmed S., Irshad L., Demirel H.O., Tumer I.Y. (2019). A comparison between virtual reality and digital human modeling for proactive ergonomic design. Proceedings of the International Conference on Human-Computer Interaction, Orlando, FL, USA, 26–31 July 2019.

[21] Bordegoni M., Cugini U., Caruso G., Polistina S. (2009). Mixed prototyping for product assessment: A reference framework. Int. J. Interact. Des. Manuf. (IJIDeM).

[22] Grazioso S., Caporaso T., Palomba A., Nardella S., Ostuni B., Panariello D., Di Gironimo G., Lanzotti A. Assessment of upper limb muscle synergies for industrial overhead tasks: A preliminary study. Proceedings of the 2019 II Workshop on Metrology for Industry 4.0 and IoT (MetroInd4.0&IoT).

[23] Merletti R., Rau G., Disselhorst-Klug C., Stegeman D., Hagg G. SENIAM Project. http://www.seniam.org/.

[24] Kirtley C. (2006). Clinical Gait Analysis: Theory and Practice.

[25] Hancock P.A., Meshkati N. (1988). Human Mental Workload.

[26] Lawson G., Salanitri D., Waterfield B. (2016). Future directions for the development of virtual reality within an automotive manufacturer. Appl. Ergon..

[27] Caporaso T., Grazioso S., Gironimo G.D., Lanzotti A. Design of Wearables for Biosignal Acquisition: A User Centered Approach for Concept Generation and Selection. Proceedings of the International Conference on Design, Simulation, Manufacturing: The Innovation Exchange.

